# Copper enhances tetracycline resistance via the efflux transporter CrdAB-CzcBA in *Helicobacter pylori*

**DOI:** 10.3389/fmed.2025.1552537

**Published:** 2025-07-21

**Authors:** Fanglin Gao, Wanquan Xiang, Xiaoyan Zhang, Xiaoxing Huang, Feifei She, Yancheng Wen

**Affiliations:** ^1^Key Laboratory of Gastrointestinal Cancer (Fujian Medical University), Ministry of Education, Fuzhou, China; ^2^Fujian Key Laboratory of Tumor Microbiology, Department of Medical Microbiology, Fujian Medical University, Fuzhou, China

**Keywords:** *H. pylori*, copper, efflux transporter, CrdAB-CzcBA, tetracycline resistance

## Abstract

*Helicobacter pylori* infection is a significant risk factor for various gastrointestinal diseases, while the standard triple therapy for its eradication is increasingly compromised by antibiotic resistance. This study investigates the role of the CrdAB-CzcBA efflux pump and its regulation by copper in tetracycline resistance in *H. pylori*. Using minimum inhibitory concentration (MIC) determination and growth curve analysis, we found that the deletion of *crdA* or *czcA* significantly reduced tetracycline resistance, while overexpression of CrdAB-CzcBA under the urease promoter enhanced bacterial resistance by reducing intracellular tetracycline accumulation. Ethidium bromide and tetracycline accumulation assays confirmed that CrdAB-CzcBA mediates active efflux of tetracycline, contributing to reduced intracellular drug levels. Furthermore, copper supplementation upregulated the expression of CrdAB-CzcBA via the CrdRS two-component system, thereby promoting bacterial growth under tetracycline stress. Notably, copper-induced resistance was abrogated in Δ*crdR* mutants, demonstrating the dependence of this mechanism on CrdRS. These findings highlight CrdAB-CzcBA as a critical efflux system in tetracycline resistance and emphasize the role of environmental factors, such as copper, in modulating bacterial antibiotic resistance, underscoring the need for strategies that account for metal ion influences in managing *H. pylori* infections.

## 1 Introduction

Gastric cancer causes more than 720,000 deaths per year worldwide and has become a global health threat (1). Infection with *Helicobacter pylori*, a Gram-negative human pathogen that infects approximately 50% of the world’s population, is a major risk factor for gastric cancer. *H. pylori* is closely related to the development of gastrointestinal diseases, including gastritis, peptic ulcers, gastric cancer, and mucosa-associated lymphoid tissue lymphoma (MALT) (2). To eradicate *H. pylori*, a standard triple therapy is recommended using a proton pump inhibitor and two of five antibiotics, including amoxicillin, tetracycline, clarithromycin, metronidazole, and levofloxacin (3–5). Currently, the commonly recommended first-line regimen is bismuth quadruple therapy, which includes tetracycline. Thus, the effectiveness of this regimen may be affected by tetracycline efflux mechanisms such as CrdAB-CzcBA. However, the eradication rate has been challenged owing to the increasing drug resistance rate worldwide (6).

Tetracycline acts as a protein synthesis inhibitor of both gram-positive and gram-negative bacteria by binding to the 30S subunit of ribosomes, thus inhibiting aminoacyl-tRNA binding (7). Tetracycline resistance is mainly caused by alterations in 16S rRNA. For clinically isolated tetracycline-resistant *H. pylori* strains, an AGA to TTC (926–928) triple-base-pair mutation preferentially occurs, whereas single or two-base-pair substitution induces only moderate levels of resistance (8). A study also showed that AGA to TTC (965–967) mutations also caused high levels of tetracycline resistance in *H. pylori* clinical isolates (9). However, some clinical isolates containing no mutations in 16S rRNA genes also showed tetracycline resistance, suggesting that other mechanisms might also be involved in tetracycline resistance in *H. pylori* (10).

In some gram-negative bacteria, tetracycline resistance is induced through TetA, an efflux protein (11, 12). TetA expression is induced by tetracycline through the transcriptional regulator TetR, which acts by directly binding to the promoter of the *tetA* gene (13). In *H. pylori*, the protein sequence of HP1165 showed similarity to the TetA of *Clostridium perfringens*, and is involved in intermediate resistance and inducible tetracycline resistance (14). However, if other efflux pumps are involved in tetracycline resistance, further investigation is required.

Drug resistance of pathogens can be either acquired or intrinsic. There are various intrinsic drug-resistant strategies, and efflux pumps play an important role in the drug resistance of gram-negative bacteria (15). There are five categories of efflux pumps: the major facilitator superfamily (MFS), the ATP-binding cassette (ABC) superfamily, the multidrug and toxic compound extrusion (MATE) family, the small multidrug resistance (SMR) family, and the resistance-nodulation-division (RND) superfamily (16, 17). The RND family has been shown to be involved in multidrug resistance of gram-negative bacteria (18, 19). The RND efflux pump is a protein complex consisting of three proteins: bacterial plasma membrane active transporters, membrane fusion proteins, and outer membrane factors. RND efflux pumps, such as MexAB-Oprm in *Pseudomonas aeruginosa* and AcrAB-TolC in *Escherichia coli* have been thoroughly studied previously (20–22). It has been reported that there are only four TolC homologous genes, including HP0605, HP0971, HP1327, and HP1489 in the *H. pylori* 26,695 genome, comprising four RND efflux systems: HP0605-0607, HP0971-0969, HP1326-1329 and HP1487-1489 (23, 24). The relationship between these efflux systems and drug resistance has been studied in different groups (23, 25–27).

CrdAB-CzcBA (HP1326-1329) comprises an RND efflux pump in *H. pylori* and is involved in copper extrusion. It consists of four main components: HP1326 (CrdA), a secreted protein crucial for maintaining cytoplasmic copper homeostasis; HP1327 (CrdB), a putative outer membrane protein believed to function as the efflux channel for metal ions; HP1328 (CzcB homolog), an inner membrane protein likely involved in substrate recognition and transport across the inner membrane; and HP1329 (CzcA homolog), another inner membrane protein thought to be responsible for the active transport of copper, possibly using the proton motive force. CrdAB-CzcBA is required for resistance to high concentrations of copper (28). The expression of CrdAB-CzcBA is induced by copper through a two-component CrdRS system (29). Copper ions play an important role in bacterial metabolism; for example, copper acts as a cofactor of enzymes involved in superoxide dismutase and cytochrome c oxidase (30, 31). However, copper is required at a low concentration, while excess copper is toxic to bacteria by generating reactive oxygen species, thus causing cellular damage (30). Copper plays an important role in the pathogenesis of *H. pylori*, and studies have shown that it promotes *H. pylori* colonization of the gastric mucosa, while copper toxicity is also employed by macrophages to eliminate bacteria through phagosomes (32, 33). In this study, we have shown here that CrdAB-CzcBA is involved in the efflux activity of extruding tetracycline in *H. pylori* and, consequently, the resistance to tetracycline. We have also found that copper enhances the resistance of *H. pylori* to tetracycline through activation of CrdAB-CzcBA.

## 2 Materials and methods

### 2.1 Strains and growth conditions

*H. pylori* strains, including Hp26695, and the clinical isolates HpFZ068 and HpFZ169, were used in this study. *H. pylori* strains were cultured under a microaerobic environment (5% O_2_, 10% CO_2_, 85% N_2_) at 37°C. Bacteria were cultured on Columbia blood agar plates (OXOID, Thermo Fisher Scientific, UK) containing 5% sheep blood or in Brucella broth (Becton Dickinson, Sparks, MD, USA) supplemented with 10% fetal bovine serum (FBS, PAN-Biotech, Aidenbach, Germany) (BB + FBS) with agitation at a speed of 120 rpm. Kanamycin (MP Biomedicals, LLC, USA) (10 μg/mL) and chloramphenicol (MP Biomedicals, LLC, USA) (20 μg/mL) were supplied as needed. *E. coli* DH5α was grown in Luria-Bertani medium at 37°C.

### 2.2 Construction of Δ*czcA*, Δ*crdA* and Δ*crdR* isogenic mutant strains of *H. pylori*

To construct a Δ*czcA* isogenic mutant of *H. pylori* 26,695, primers CzcA-upF and CzcA-upR were used to amplify the upstream sequence of *czcA* from *H. pylori* 26,695 genomic DNA. Primers CzcA-downF and CzcA-DownR were used to amplify the downstream sequence of *czcA*. The kanamycin-resistant genes were amplified using the primers AphA-F and AphA-R. PCR reactions were performed using PrimeSTAR HS DNA Polymerase (Takara, Beijing, China). These DNA fragments were cloned into the pBluescript SK II (−) vector (Stratagene, La Jolla, CA, USA) using the ClonExpress^®^ Ultra One Step Cloning Kit (Vazyme, Nanjing, China), generating pBlue-CzcAKO, which was then transformed into *E. coli* DH5α. After confirmation by colony PCR and DNA sequencing, pBlue-CzcAKO was transformed into *H. pylori* 26,695 by electroporation. Bacteria were selected using Columbia blood agar plates containing kanamycin (3 μg/mL), and Δ*czcA* was confirmed by colony PCR and DNA sequencing. CzcA-upF, CzcA-upR, CzcA-downF, and CzcA-downR were used for the construction of HpFZ068Δ*czcA* and HpFZ169Δ*czcA*, and the experiments were performed as described above. To construct Δ*crdR*, primers CrdR-upF, CrdR-upR, CrdR-downF, and CrdR-downR were used. To construct Δ*crdA*, primers CrdA-upF, CrdA-upR, CrdA-downF, and CrdA-downR were used. Primers used in this study were listed in Supplementary Table 1.

### 2.3 Construction of *crdAB*-*czcBA*^he^, crdAB-ΔczcA^he^ and Hp26695^chl^

To facilitate the substitution of the *crdAB*-*czcBA* promoter with the ureAB promoter, the urease promoter fragment was obtained via amplification with primers P*ureAB*-F and P*ureAB*-R. Concurrently, the chloramphenicol resistance cassette (CAT) was amplified using primer pairs ChlR-F and ChlR-R. DNA segments harboring the upstream region of the *crdA* promoter were amplified with the primer pairs CrdA^he^-upF and CrdA^he^-upR, whereas downstream sequences were amplified using CrdA^he^-downF and CrdA^he^-downR. These fragments were subsequently ligated into the pBluescript SK II (−) vector, resulting in the construction of pBlue-CrdA^he^. This construct was then introduced into *H. pylori* 26,695 and Δ*czcA* strains and selected on Columbia blood agar plates containing chloramphenicol (4 μg/mL), yielding the *crdAB*-*czcBA*^he^ and *crdAB*-Δ*czcA*^he^ strains, respectively. For the generation of chloramphenicol-resistant Hp26695 (Hp26695^chl^), the upstream and downstream sequences flanking the *crdA* promoter were amplified using the primer sets CrdA^he^-upF, CrdA^he^-upR1, CrdA^he^-downF1, and CrdA^he^-downR, and the CAT was amplified with Chl^R^-F and Chl^R^-R. These amplified products were cloned into pBluescript SK II (−), creating pBlue-Chl^R^, which was subsequently transformed into *H. pylori* 26,695. Primers utilized in this study are itemized in Supplementary Table 1.

### 2.4 Determination of antibiotic susceptibility

*H. pylori* wild type strain and clinical isolates were cultivated on Columbia Blood agar plates containing 5% sheep blood for 3 days. Then the bacterial cells were collected and resuspended in Brucella broth, and 100 μL of cell suspension with 2 McFarland standard were spread onto Mueller-Hinton agar plates (OXOID, Thermo Fisher Scientific, UK) containing 5% sheep blood. The minimum inhibitory concentration (MIC) of each strain to amoxicillin, tetracycline, clarithromycin, metronidazole, and levofloxacin were determined by MIC test strips (MTS, Liofilchem, Italy) which were placed onto the inoculated plates. The plates were incubated at 37°C under microaerobic conditions, and the MIC values were determined after 72 h. Each MIC value represents the average from three independent experiments.

### 2.5 RNA extraction, reverse transcription and qPCR assay

To quantify the expression of efflux pumps stimulated by copper, an overnight culture of *H. pylori* 26,695 was resuspended in BB + FBS with an initial OD_600_ of 0.2, with or without 50 μM CuSO_4_. Bacterial culture was maintained for 4 h and RNA was extracted as described above. All samples were analyzed in at least three biological replicates. Bacterial RNA was extracted using the TRIzol^®^ Reagent (Life Technologies, Grand Island, NY, USA) according to the manufacturer’s protocol, and reverse transcription was performed using the HiScript^®^II Q RT SuperMix for qPCR kit (Vazyme, Nanjing, China) with 0.5 μg of RNA in a 20 μL reaction system. For quantitative real-time PCR (qPCR), SYBR qPCR Master Mix (Vazyme, Nanjing, China) was used, and the primers for each gene are listed in Supplementary Table 1. The 16S rRNA amplicon was used as an endogenous control, and relative mRNA levels were determined using the 2^−ΔΔCt^ method.

### 2.6 Monitoring the growth of *H. pylori* influenced by etracycline and copper

To monitor the growth curves of *H. pylori*, bacteria were first cultured for 24 h in BB + FBS. Then, bacterial cells were resuspended in fresh BB + FBS at an initial OD_600_ of 0.1, with or without supplementation of antibiotics or CuSO_4_. Tetracycline (0.023 μg/mL) was supplemented at the subinhibitory concentration (0.5 × MIC), and 50 μΜ CuSO_4_ was supplemented as indicated. Bacterial culture was maintained for 48–72 h, and the OD_600_ of the culture was monitored at the indicated time points. Each experiment was independently performed at least three times.

### 2.7 Ethidium bromide accumulation assay and tetracycline accumulation assay

Ethidium bromide (EB) accumulation assay was performed as previously described (25). In brief, *H. pylori* cells were resuspended in fresh BB + FBS with an initial OD_600_ of 0.1 and cultured in BB + FBS until the exponential phase (OD_600_ = 0.6). The cells were subsequently washed twice with PBS (pH = 7.0), and the bacterial cells were harvested and resuspended in 100 μL PBS (OD_600_ = 0.4) in a 96-well plate (Corning3603, Carlsbad, CA, USA). Then, 100 μL of EB was added at a final concentration of 10 μg/mL. Fluorescence was measured using an EnSight™ Multimode Plate Reader (PerkinElmer, Waltham, MA, USA) under room temperature, with an excitation wavelength of 530 nm and an emission wavelength of 590 nm (34). Luminescence was recorded every 60 s for 30 min. Each experiment was performed in triplicate, and values are shown as the averages of three independent experiments.

For the tetracycline accumulation assay, the experiments were performed as described previously (35). Briefly, *H. pylori* strains were cultured overnight in BB + FBS until the exponential growth phase. Then, 0.8 × 10^9^ cells/sample were washed twice with 2 mL of Mg^2+^ buffer (50% methanol, 10 mM Tris–HCl, 0.1 mM MgCl_2_, and 0.2% glucose, pH 8.0), and the bacterial cells were resuspended in 2 mL Mg^2+^ buffer containing tetracycline (100 μg/mL) and incubated for 15 min (36). After centrifugation, the cells were collected and resuspended in 2 mL Mg^2+^ buffer. For measurement of tetracycline accumulation, 100 μL of each sample was added to a 96-well black plate (Corning, Carlsbad, CA, USA), and fluorescence was measured using an EnSight™ Multimode Plate Reader (PerkinElmer, Waltham, MA, USA) under room temperature, with excitation and emission wavelengths of 400 and 520 nm, respectively (37).

### 2.8 Statistical analysis

Data are presented as the mean ± standard error. To assess data significance, an unpaired *t-test* was used to study the degree of statistical analysis of the two groups. Statistical significance was set at *p* < 0.05. GraphPad Prism software (version 8.0) was used to analyze the results.

## 3 Results

### 3.1 CrdAB-CzcBA contributes to the tetracycline resistance of *H. pylori*

**Figure 1:**
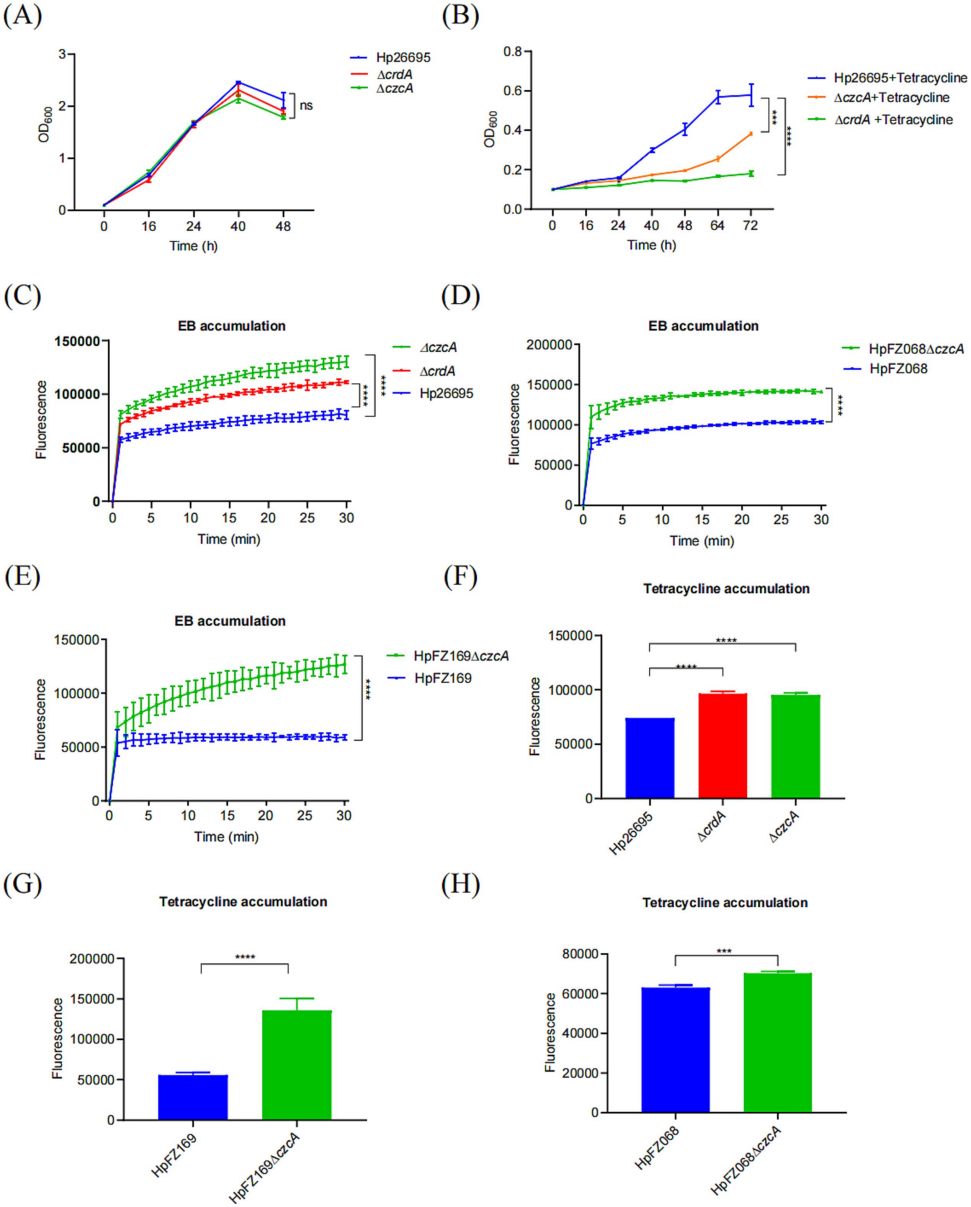
CrdAB-CzcBA contributes to the tetracycline resistance of *H. pylori.* **(A,B)** Growth curves for Hp26695, Δ*crdA*, Δ*czcA* in the presence **(A)** or with **(B)** 0.023 μg/mL tetracycline. **(C–E)** EB accumulation measured by the fluorescence intensity, indicating active efflux in Hp26695, Δ*crdA*, Δ*czcA*. Clinical isolates HpFZ068 and HpFZ169, along with their respective Δ*czcA* mutants, were included for comparison. Fluorescence intensity was recorded at 30-s intervals over 30 min. **(F–H)** Tetracycline accumulation assays performed for Hp26695, Δ*crdA*, Δ*czcA*, clinical isolates HpFZ068 and HpFZ169, and their Δ*czcA* mutants. Each value represents the mean ± standard deviation from three independent experiments, and error bars represent the standard deviation. Statistical significance is indicated as ***p < 0.001; ****p < 0.0001; ns, non-significance.

To verify whether the RND efflux pump CrdAB-CzcBA is involved in drug resistance, we constructed isogenic Δ*crdA* and Δ*czcA* mutants. We investigated the MICs of antibiotics in *the H. pylori* wild type, Δ*crdA*, Δ*czcA* (Table 1; Supplementary Table S2). We found that MIC values of levofloxacin, metronidazole, clarithromycin and amoxicillin showed no significant difference between the wild type strain and mutant strains. However, the MIC of tetracycline in *H. pylori* Δ*crdA* and Δ*czcA* was 0.016 mg/L, compared to 0.047 mg/L in the wild type strain Hp26695. This reduction was also observed in clinical isolates: the MIC of HpFZ068Δ*czcA* was 0.032 mg/L versus 0.125 mg/L in the parental strain HpFZ068; and for HpFZ169Δ*czcA*, the MIC was 0.04 mg/L compared to 0.08 mg/L in the parental strain HpFZ169. To confirm this result, we measured the growth curves of *H. pylori* 26,695 wild type, Δ*crdA*, and Δ*czcA* mutants cultivated in Brucella broth with or without tetracycline (0.023 mg/L, 0.5 × MIC). Knockout of *crdA* or *czcA* had no effect on the growth of the wild type *H. pylori* in the absence of antibiotics (Figure 1A). In the presence of tetracycline, the growth of *H. pylori* strains was attenuated, however, Δ*crdA* and Δ*czcA* exhibited a higher sensitivity to tetracycline compared to the wild type, with a more retarded growth (Figure 1B). These results certificated that CrdAB-CzcBA is involved in tetracycline resistance in *H. pylori*.

**Table 1:** Minimum inhibitory concentrations (MICs) for tetracycline against *Helicobacter pylori.*

Strains	MIC (mg/L)
Hp26695	0.047
Δ*crdA*	0.016
Δ*czcA*	0.016
Hp26695 + CuSO_4_	0.125
Δ*crdA+*CuSO_4_	<0.016
Δ*czcA+*CuSO_4_	<0.016
HpFZ068	0.125
HpFZ068Δ*czcA*	0.032
HpFZ169	0.08
HpFZ169Δ*czcA*	0.04
Hp26695^chl^	0.047
*crdAB*-*czcBA*^he^	0.19
*crdAB*-Δ*czcA*^he^	0.032

To further investigate the role of CrdAB-CzcBA in tetracycline resistance within *H. pylori*, efflux activity was assessed through ethidium bromide (EB) accumulation assays. These assays revealed increased EB accumulation in the Δ*czcA* and Δ*crdA* mutants compared to the wild type, indicating a deficit in efflux activity (Figure 1C). This pattern was also observed in two additional clinical isolates, HpFZ068 and HpFZ169, where the Δ*czcA* mutants accumulated more EB than their respective parent strains (Figures 1D,E). To more directly assess tetracycline efflux activity, we performed tetracycline accumulation assays. Similarly, the Δ*crdA* and Δ*czcA* strains accumulated greater amounts of tetracycline than the wild type, suggesting CrdAB-CzcBA is involved in tetracycline efflux (Figure 1F). These findings were consistent with the clinical isolate strains, which demonstrated increased tetracycline accumulation in the Δ*czcA* mutants compared to their parental strains (Figures 1G,H), supporting the role of CrdAB-CzcBA in mediating tetracycline efflux and contributing to resistance by diminishing intracellular antibiotic concentrations.

### 3.2 Elevated expression of CrdAB-CzcBA significantly enhanced tetracycline resistance

**Figure 2:**
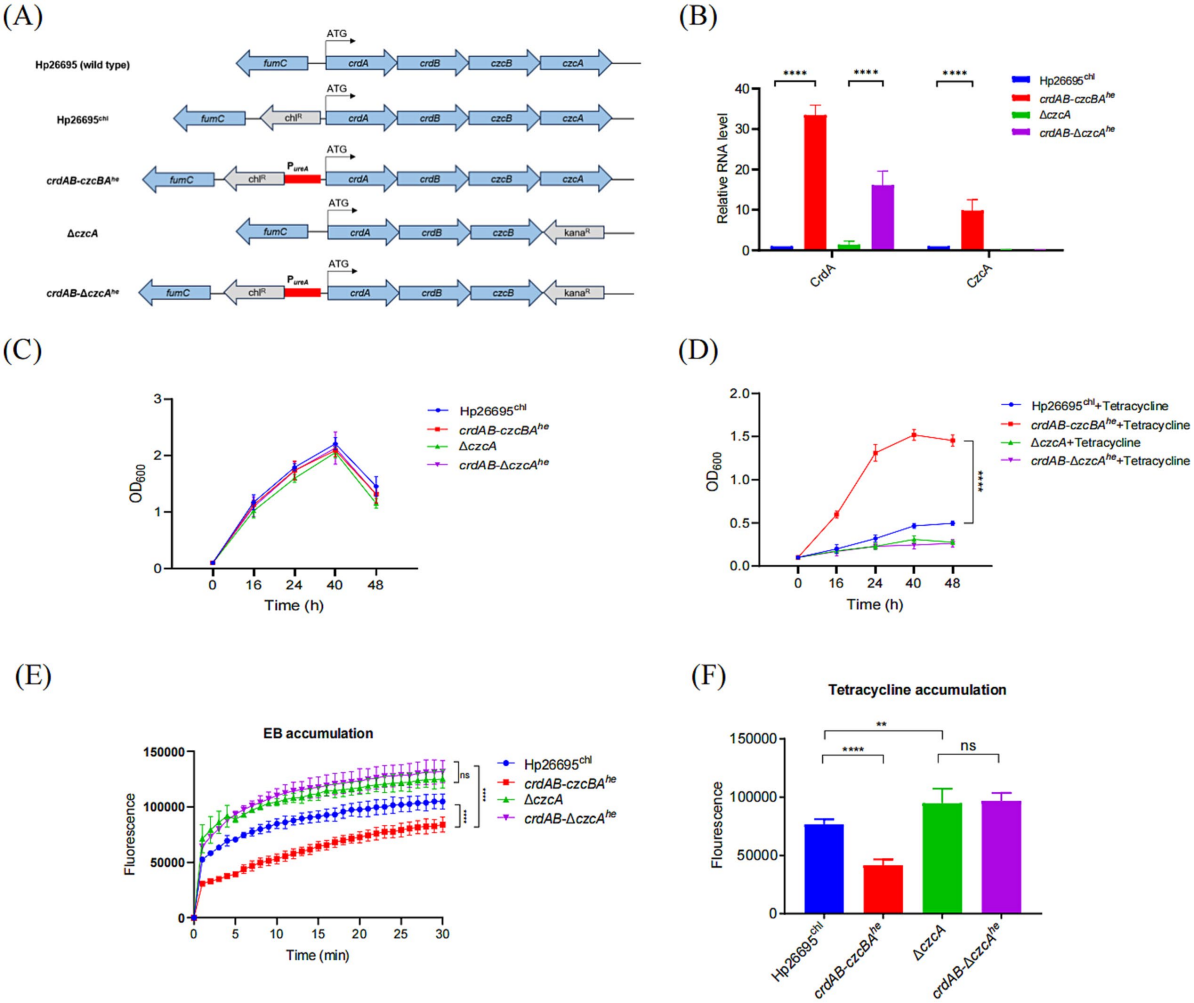
Overexpression of CrdAB-CzcBA enhances resistance to tetracycline. **(A)** Schematic representation of the constructs for high-expression strains derived from Hp26695, utilizing the urease promoter region (red bar), antibiotic resistance genes (gray), and *H. pylori* genes (blue). Start codon is indicated by arrows. **(B)** qPCR quantification of CrdA and CzcA mRNA in Hp26695^chl^, crdAB-czcBA^he^, Δ*czcA*, and crdAB-Δ*czcA*^he^, normalized to Hp26695^chl^. **(C,D)** Growth profiles in the absence **(C)** and presence **(D)** of tetracycline. **(E,F)** EB accumulation assays **(E)** and tetracycline accumulation assays **(F)** performed for Hp26695^chl^, Δ*czcA*, overexpression strains crdAB-czcBA^he^ and crdAB-Δ*czcA*^he^. Each value represents the mean ± standard deviation of three independent experiments. Error bars represent standard deviation. Statistical significance is indicated as **p < 0.01, ***p < 0.001, ****p < 0.0001; ns, non-significance.

A previous study comparing the MIC difference between Δ*czcA* and wild type suggested that the efflux pump CrdAB-CzcBA was not involved in antibiotic resistance in *H. pylori* 1,061 strain (23). However, we suspected this might be due to its low expression level under normal laboratory culture condition. So we replaced its native promoter with the robust urease (coded by *ureAB*) promoter in both Hp26695 wild type and Δ*czcA* strains, hypothesizing that augmented expression would correlate with increased resistance, aiming to provide a direct link between the overexpression of CrdAB-CzcBA efflux pump genes and enhanced antibiotic resistance phenotypes (Figure 2A). The increased expression levels of CrdA and CzcA were verified, with marked elevation under the *ureAB* promoter compared to the Hp26695 strain containing only the chloramphenicol resistance gene (Figure 2B). Furthermore, the overexpression of CrdAB-CzcBA did not significantly affect bacterial growth under normal cultivation conditions (Figure 2C). When challenged with tetracycline, CrdAB-CzcBA overexpression substantially improved bacterial growth, an effect not observed in the Δ*czcA* background (Figure 2D). We have also confirmed this result by comparing the MICs of Hp26695^chl^, *crdAB*-*czcBA*^he^ and *crdAB*-Δ*czcA*^he^ strains (Table 1). The results showed that MIC of tetracycline in *H. pylori crdAB*-*czcBA*^he^, but not *crdAB*-Δ*czcA*^he^, was 4-fold higher than that in Hp26695 wild type strain containing chloramphenicol resistance cassette (Hp26695^chl^). Further analysis of EB accumulation and tetracycline accumulation showed that strains overexpressing *crdAB-czcBA* (*crdAB*-*czcBA*^he^) showed reduced EB and tetracycline accumulation levels compared to the control Hp26695^chl^ strain, yet there was no significant difference between the *crdAB*-Δ*czcBA*^he^ strain and the Δ*czcA* strain (Figures 2E,F), certified that induction of CrdAB-CzcBA resulted in the significant increase in its efflux capacity. Collectively, these data certified that when expression of CrdAB-CzcBA was activated, *H. pylori* showed enhanced efflux capacity and resistance to tetracycline.

### 3.3 Copper enhances bacterial growth under tetracycline in *H. pylori*

**Figure 3:**
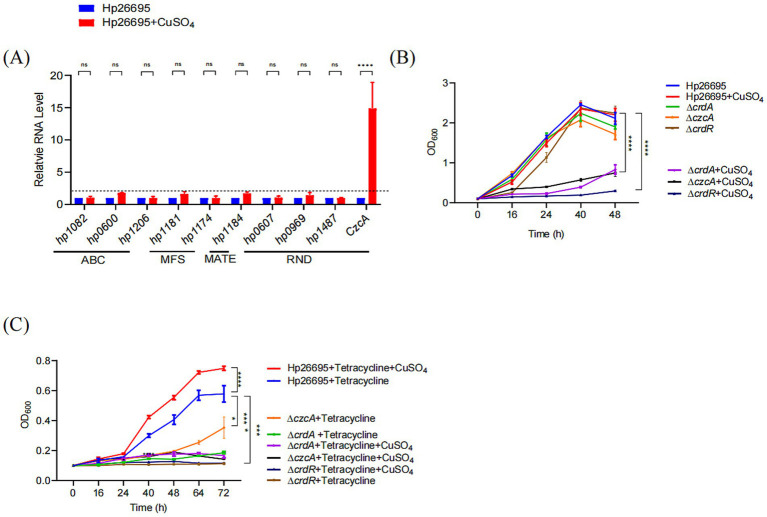
Copper enhances the bacterial growth under tetracycline in *H. pylori*. **(A)** Copper modulates expression levels of *H. pylori* efflux pump-related genes. The expression of efflux pumps was quantified by qPCR with mRNA levels normalized to those in the untreated Hp26695 control. The dashed line represents a one-fold increase in relative expression due to copper supplementation. **(B)** Growth curves for Hp26695, Δ*crdA*, Δ*czcA* and Δ*crdR* with or without supplementation of 50 μM CuSO_4_. Data points represent the mean OD_600_ values from three independent experiments with standard deviations indicated. Statistical significance: ****p < 0.0001. **(C)** Growth curves for Hp26695, Δ*crdA*, Δ*czcA* and Δ*crdR* under 0.023 μg/mL tetracycline, with or without supplementation of 50 μM CuSO_4_. Data points reflect the mean OD_600_ values from three independent experiments, with standard deviations indicated. Statistical significance: ****p < 0.0001.

The expression of CrdAB-CzcBA is induced by copper, we then confirmed this result, showing that the expression of CzcA was greatly enhanced by copper with expression upregulated more than 14-folds (Figure 3A). We have also investigated the expression of the nine genes representing all the other efflux pumps reported in *H. pylori*, including ABC transporter family proteins (HP1206, HP1082, and HP0600) (38), MFS family proteins (HP1181and HP1174) (39, 40), MATE family protein (HP1184) (23), and RND family proteins (HP0607, HP0969, HP1487). The expression of HP1082, HP1206, HP1174, HP0607, HP0969, and HP1487 was not influenced by copper, while the expression of HP0600, HP1181, and HP1184 was 78, 95, and 71% higher, respectively, in the presence of copper. These results suggest that only CrdAB-CzcBA is significantly activated by copper. To verify whether copper enhances tetracycline resistance through activating expression of CrdAB-CzcBA, we cultivated *H. pylori* 26,695, Δ*crdA*, and Δ*czcA* mutants in the presence or absence of 50 μM CuSO_4_ and tetracycline. As expected, supplementation with 50 μM CuSO_4_ inhibited the growth of the Δ*crdA* and Δ*czcA* strain but had no effect on growth of the wild type strain (Figure 3B). In the presence of tetracycline, the addition of copper promoted the growth of wild type strain, but had no effect on the growth of Δ*crdA*. Compared to Δ*crdA*, *H. pylori* mutant Δ*czcA* showed a similar phenotype, i.e., Δ*czcA* was more sensitive to tetracycline than the wild type strain, and copper inhibited the growth of Δ*czcA* in the presence or absence of tetracycline (Figure 3C). These results suggest that copper induced tetracycline resistance through activation of CrdAB-CzcBA. CrdRS is a two-component system responsible for sensing copper and the activation of CrdAB-CzcBA (29). To prove that copper-induced tetracycline resistance was dependent on CrdRS, we constructed a Δ*crdR* strain and evaluated its resistance to tetracycline in the presence or absence of copper. As previously reported, copper inhibited the growth of Δ*crdR* (Figure 3B). Growth of Δ*crdR* is significantly inhibited by tetracycline compared to the *H. pylori* wild type strain, suggesting that CrdR contributes to tetracycline resistance. Tetracycline resistance was not promoted by copper in Δ*crdR*, suggesting that copper-induced tetracycline resistance is dependent on CrdR (Figure 3C). These results suggest that copper induces the expression of CrdAB-CzcBA through CrdR, enhancing bacterial resistance to tetracycline.

## 4 Discussion

Efflux pumps provide intrinsic antibiotic resistance to bacteria and are thus considered therapeutic targets for the mediation of antibiotic resistance. Several methods have been proposed to inhibit the function of efflux pumps, including downregulation of their expression by interfering with the regulator system, directly inhibiting the assembly or action of these pumps, or modification of antibiotics so that they can no longer act as substrates of efflux pumps (41, 42). However, efflux pumps are present in both drug-sensitive and drug-resistant strains (15). Environmental cues that stimulate the expression of efflux pumps may lead to higher resistance to the corresponding antibiotics. In this study, we found that the copper resistance determinants CrdAB-CzcBA are involved in tetracycline resistance. Unlike some efflux pumps such as MexAB-OprM and NorA, which are involved in the efflux of distinct classes of drugs, and substrates, CrdAB-CzcBA showed no significant effect on resistance to antibiotics, including levofloxacin, metronidazole, clarithromycin, and amoxicillin (data not shown) (15, 42). This is also supported by the finding that copper showed no cross-protection of *H. pylori* to these antibiotics, which also failed to stimulate the expression of the CrdAB-CzcBA operon (data not shown). This suggests that the CrdAB-CzcBA efflux pump only extrudes specific antibiotics. This is also the case for efflux systems such as AbaF, which provide resistance to Fosfomycin (43). Efflux in enteric rods can also promote bile resistance, suggesting a complex role of these pumping systems (44). If CrdAB-CzcBA is involved in the resistance of other substrates, further investigation is required.

Tetracyclines inhibit protein translation by interfering with bacterial ribosomes and are widely used in both human medicine and livestock production worldwide. Approximately 11 classes, including more than 40 genes, have been characterized as tetracycline-resistant genes. Among these, approximately 60 percent are involved in efflux pumps by extruding tetracycline extracellularly with substrate specificity (12, 45, 46). All of these genes belong to the MFS family, which are single polypeptides, and are proton motive force-dependent (47, 48). In *H. pylori*, only HP1165 was shown to be involved in induced tetracycline resistance (14). Several studies have shown that knockout of efflux pumps in *H. pylori* does not alter tetracycline resistance (23). We suspect that this might be due to a relatively low level of expression of these genes *in vitro*. A higher expression level of these efflux pump genes *in vivo* might play a significant role in antibiotic resistance. One *in silico* study also found that there are 27 genes in *H. pylori* that encoding putative translocases belonging to the ABC transporter, MAT, MFS, and RND families (23). More genes involved in antibiotic resistance require further investigation.

Copper enhances the resistance of tetracycline by enhancing the expression of CrdAB-CzcBA through the two-component CrdRS system (Figure 3) (28, 29). This finding is significant as it reveals a previously unrecognized link between metal ion homeostasis and antibiotic resistance in *H. pylori*. Furthermore, while similar copper-induced efflux systems have been described in other bacteria, such as the CzcCBA system in *P. aeruginosa*, these systems are primarily associated with resistance to different antibiotics and heavy metals (49, 50). To our knowledge, this is the first report of copper-induced tetracycline resistance via an RND efflux pump in *H. pylori*. Besides, other factors that regulate CrdRS activity might also result in the alteration of CrdAB-CzcBA expression. Studies have shown that CrdRS-CrdA is important for survival under nitrosative stress, and the expression of CrdA is activated by CrdRS in response to nitric oxide (51). This suggests that nitrosative stress, such as that occurring during inflammation of the stomach, may alter the resistance to copper and tetracycline.

Contrary to prior observations in *H. pylori* strain 1,061 that discounted the role of CrdAB-CzcBA in antibiotic resistance (23), our data indicate a substantial increase in tetracycline resistance upon overexpression of CrdAB-CzcBA. This discrepancy could be attributed to the relatively low expression of CrdAB-CzcBA under standard laboratory conditions, which may mask its role in resistance. The concept that efflux pumps with low baseline expression can exhibit a pronounced resistance phenotype upon activation is supported by findings in *E. coli*, where the overexpression of typically lowly expressed RND family efflux pumps, such as *yhiUV* (52), has been linked to increased resistance against a range of antibiotics including fluoroquinolones, linezolid, and tetracycline. Hence, the functional impact of efflux pumps expressed at low levels under basal conditions may become more apparent upon induction, underlining the potential for adaptive resistance mechanisms.

*H. pylori* survives in the stomach environment of humans and needs to respond to environmental signals, such as pH changes, nutrient limitation, and reactive oxygen species. Transition metals participate in various processes, including acting as nutrients for living organisms by incorporation into metalloproteins (53, 54). The host limits the availability of these metals to bacteria through nutritional immunity (55). Copper has been utilized by many bacteria as a cofactor for enzymes, including superoxide dismutase and NADH dehydrogenase (30, 31). However, excess copper can cause the generation of reactive oxygen species, including superoxide radicals, through Fenton reactions, and can thus damage cellular macromolecules and cellular structures (56, 57). Our study suggests that copper homeostasis is closely related to the survival and drug resistance of the bacterium.

Environmental factors can drastically alter the expression of specific genotypes of bacteria, conferring antibiotic resistance. One study showed that *Salmonella Typhimurium* was found to be significantly more resistant to antibiotics when grown in an environment mimicking conditions under low pH, magnesium, and phosphate compared to grown in standard media (58). Other studies have also shown that environmental conditions that reduce the growth rate activate the drug resistance gene through a stringent response (59). The expression of CrdAB-CzcBA is silenced under normal laboratory conditions, whereas copper significantly activates its expression, suggesting that environmental signals are strongly correlated with the drug resistance of the bacterium. However, using a standard medium might fail to elucidate the role of some silenced genes involved in antibiotic resistance (28, 29). More host environmental factors involved in modulating the bacterial resistance to antibiotics deserve further investigation. Copper concentration in serum is up to 1.5 mg/L (23.6 μM) in healthy individuals (60). However, copper is important in the inflammatory response for its bactericidal effect against pathogen (61). Significant copper accumulation was found both in the serum and tissue during inflammation, this suggests that *H. pylori* infection which causes gastritis might lead to the activation of CrdAB-CzcBA expression (62, 63). High expression of efflux pumps plays an important role in clinical drug-resistant isolates, and this might be due to the mutations in the promoter region or in the regulatory proteins (15). We speculate that mutation in promoter of *crdAB*-*czcBA* or in CrdRS that resulted in activation of CrdAB-CzcBA might lead to a significant resistance to tetracycline in clinical isolates of *H. pylori*.

## 5 Conclusion

Taken together, our results showed that CrdAB-CzcBA comprises an efflux pump, with tetracycline and EB efflux activity, and is involved in tetracycline resistance. Copper activated CrdAB-CzcBA expression by acting on CrdRS, increasing bacterial resistance to tetracycline in *H. pylori*. Our study suggests that copper is an important nutrient for bacteria and plays a role in the cross-protection of tetracycline resistance.

## Data Availability

The original contributions presented in the study are included in the article/Supplementary material, further inquiries can be directed to the corresponding authors.
