# Supplementary material for: Copper enhances tetracycline resistance via the efflux transporter CrdAB-CzcBA in Helicobacter pylori
Source: Front Med (Lausanne). 2025 Jul 21;12:1552537. doi: 10.3389/fmed.2025.1552537 (PMC12319023; doi:10.3389/fmed.2025.1552537)
Supplement: Supplementary file 1 [file Data_Sheet_1.pdf]

Supplementary table 1. Primers used in this study

Primer name	Sequence (5' to 3')
	Construction of Δ*crdA*
CrdA-upF	ctatagggcgaattgggtaccAGCACGCTCACAATGTGCATT
CrdA-upR	ataaaaccgcccagtTGCTCCCATGCGTTTAAACC
CrdA-downF	cttcatttttaatttGTGGATATTAAATCTAAAGAGGGTCAGG
CrdA-downR	caggaattcgatatcaagcttTTCACCCATAGAATAATGGCTGG
AphA-F	ACTGGGCGGTTTTATGGACA
AphA-R	AAATTAAAAATGAAGTTTTAGCACGTG
	Construction of Δ*czcA*
CzcA-upF	ctatagggcgaattgggtaccAAACTCTAGCATTGAAAAAATCATTAGC
CzcA-upR	gcccagtCAAAGAGGCTAAAAAAATGAGTAGAGTG
CzcA-downF	TTTTAACGCTTTTTATTATCCCTACG
CzcA-downR	caggaattcgatatcaagcttCTTACTCAACCACTCTTATTGGATTTTT
	Construction of Δ*crdR*
CrdR-upF	ctatagggcgaattgggtaccAGCTTGAGAAAAAATCTTTTTGTGG
CrdR-upR	aaaccgcccagtAGGTAATCGTCTTCTAGTAAAAAAATCTTT
CrdR-downF	TTTGGGTAAAAATTGCATAGAAACG
CrdR-downR	caggaattcgatatcaagcttCGTTAGCTCTATGTGGATATACCCA
	Construction of *crdAB-czcAB*^{he}, *crdAB*- Δ *czcA*^{he} and Hp26695^{chl}
CrdA^{he}-upF	ctatagggcgaattgggtaccGTCTGTGCGTGATTTCTAGCACG
CrdA^{he}-upR	caacacaatatggcggaCATTAAGATAACAAGAAAATTCTTGTTGC
Chl^{R}-F	atgTCCGCCATATTGTGTTGAAACA
Chl^{R}-R	agtgataatggGGGCACCAATAACTGCCTTAAA
P*ureAB*-F	ttggtgcccCCATTATCACTCCAATTTTAATTCTCA
P*ureAB*-R	tcgttttgacCTTATTCTCCTATTCTTAAAGTGTTTTTCC
CrdA^{he}-downF	ggagaataagGTCAAAACGATGAAAAAGTTAGCCG
CrdA^{he}-downR	caggaattcgatatcaagcttGCCTAAATACAAAATGGGGTTATCC
CrdA^{he}-upR1	aactttttcatcgttttgacGGGCACCAATAACTGCCTTAAA
CrdA^{he}-downF1	GTCAAAACGATGAAAAAGTTAGCCG
	qPCR of Efflux pump genes
Hp0607-F	CGTTACGGTGCTTGCTGAGCCTAAT
Hp0607-R	CCCGTTGCTCTCTTTGGCGTTATCC
Hp0969-F	GCGTGGATAGAGATGGCGAAACCTT
Hp0969-R	GGTGCGAGACTGGCTGATGACTT
Hp1487-F	AGACCACGACAAGCCATCAAGTAGC
Hp1487-R	CCCATACGCTTCGGCGGAGTTT
Hp1184-F	TAGCGATGGTAGCGATGCTGATTGG
Hp1184-R	CATGCCCTATCACGGTGGCTATCG
Hp0600-F	TGATGAAGCCACTTCCGCTTTAGACA
Hp0600-R	CGTGCTTGGGTTGTGCGAAATAAGAA
Hp1082-F	AGCTTAACGCTATCCACAACGGTCT
Hp1082-R	TCCCAAACAGACTCACAAGCCCTAT
Hp1181-F	GGTGGTTATGGTGTGCTTGCTGTTG

Hp1181-R	CTTCATCCGCCACCATCGCACTAAT
Hp1174-F	TGGCTACGCTCAATTTAGGGCATCT
Hp1174-R	AGCATGTCTGTAACCGCACCTTGAA
Hp1206-F	AAAGGGTGAGATGGCTGCCAAATCC
Hp1206-R	CTTGTGGGCTTCTTCGTGGTTGTCT
	qPCR of *crdAB-czcBA*
CrdA-F	CAGCGATGAAAGAAATGGCGCAAGT
CrdA-R	ATATCCACCCTAACCTGCCATGTCC
CrdB-F	ATGCTATCTTTTATAAGCGCGTTTG
CrdB-R	TCATTCTAATCCTTTAAGGTTTTGTAAAG
CzcB-F	TTACTCTCACTTCAACGGCATTATTT
CzcB-R	AATCCTCTTGATTGACTTTAACCAGC
CzcA-F	AGGAAGTGGTGGGCGGGATTGT
CzcA-R	TCGCTCCTGTCATACACGCTGGT
16S rRNA-F	CTCATTGCGAAGGCGACCT
16S rRNA-R	TCTAATCCTGTTTGCTCCCCA

The lowercase letters represent overlapping nucleotides.
