# Supplementary material for: Copper enhances tetracycline resistance via the efflux transporter CrdAB-CzcBA in Helicobacter pylori
Source: Front Med (Lausanne). 2025 Jul 21;12:1552537. doi: 10.3389/fmed.2025.1552537 (PMC12319023; doi:10.3389/fmed.2025.1552537)

**Supplementary table 2** Minimum inhibitory concentrations (MICs) for antibiotics against *H*. *pylori*


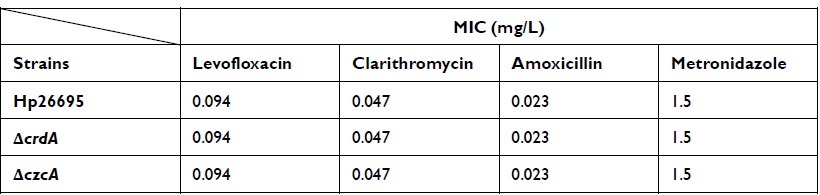

Supplement: Supplementary file 2 [file Data_Sheet_2.docx]
